# Estimation of universal and taxon-specific parameters of prokaryotic genome evolution

**DOI:** 10.1371/journal.pone.0195571

**Published:** 2018-04-13

**Authors:** Itamar Sela, Yuri I. Wolf, Eugene V. Koonin

**Affiliations:** National Center for Biotechnology Information, National Library of Medicine, National Institutes of Health, Bethesda, MD, United States of America; CPERI, GREECE

## Abstract

The results of our recent study on mathematical modeling of microbial genome evolution indicate that, on average, genomes of bacteria and archaea evolve in the regime of mutation-selection balance defined by positive selection coefficients associated with gene acquisition that is counter-acted by the intrinsic deletion bias. This analysis was based on the strong assumption that parameters of genome evolution are universal across the diversity of bacteria and archaea, and yielded extremely low values of the selection coefficient. Here we further refine the modeling approach by taking into account evolutionary factors specific for individual groups of microbes using two independent fitting strategies, an ad hoc hard fitting scheme and a mixture model. The resulting estimate of the mean selection coefficient of *s*∼10^−10^ associated with the gain of one gene implies that, on average, acquisition of a gene is beneficial, and that microbial genomes typically evolve under a weak selection regime that might transition to strong selection in highly abundant organisms with large effective population sizes. The apparent selective pressure towards larger genomes is balanced by the deletion bias, which is estimated to be consistently greater than unity for all analyzed groups of microbes. The estimated values of *s* are more realistic than the lower values obtained previously, indicating that global and group-specific evolutionary factors synergistically affect microbial genome evolution that seems to be driven primarily by adaptation to existence in diverse niches.

## Introduction

Prokaryotes have compact genomes, in terms of the number of genes and especially gene density, with typically short intergenic regions comprising less than 10% of the genome [1–3]. Deciphering the evolutionary forces that keep prokaryotic genomes compact is an important problem in evolutionary biology. The common view, steeped in a population-genetic argument, is that selection favors compact genomes in the fast-reproducing prokaryotes with large effective population sizes, to minimize the replication time and the energetic burden that is associated with gene expression [1,4]. This theory provides a plausible explanation for the observed dramatic differences in the typical size and architecture between prokaryotic and eukaryotic genomes, with the latter being up to several orders of magnitude larger than the former and, in many case, containing extensive non-coding regions [5]. Under the population-genetic perspective, the large effective population sizes of prokaryotes enhance the selection pressure and allow efficient elimination of superfluous genetic material [1,4,6,7].

The population-genetic theory predicts an inverse correlation between genome size and the strength of selection, and this prediction generally holds across the full range of genome sizes, from viruses to multicellular eukaryotes [1,6]. However, a detailed analysis of the relationship between the genome size and selection strength among prokaryotes reveals the opposite trend: genome size correlates positively and significantly with the protein-level selection strength indicating that larger genomes are typically subject to stronger selection on the protein level [8–10]. The protein-level selection is measured by the ratio of non-synonymous to synonymous mutation rates (d*N*/d*S* ratio) [11] in core genes that are common across (nearly) all prokaryotes [12]. The underlying assumption is that the effects of single non-synonymous mutations in these core, functionally conserved genes are similar (associated with similar selection coefficients) across all prokaryotes [10]. The differences in the observed d*N*/d*S* values between groups of prokaryotes are accordingly assumed to reflect differences in selection strength. At least formally, within the population-genetic theory, this assumption translates to similar selection coefficients but different effective population sizes.

Recently, we performed an analysis of the factors that govern prokaryotic genome size evolution by developing a population-genetic evolutionary model and testing its predictions against empirical genome size distributions in groups of closely related bacterial and archaeal genomes [10]. Within the modeling framework of our previous study [10], the genome size evolution is represented as stochastic gain and loss of genes, an approach that is motivated by the dominant role of horizontal gene transfer in microbial evolution [13–17]. Specifically, the model predicts a distribution of the genome sizes for the given values of the effective population size, the deletion bias and the selection coefficient associated with the gain of a gene. Using maximum-likelihood optimization methods, the values of the deletion bias and the selection coefficients can be inferred from the data. Under the simplifying assumption that the mean selection coefficients and deletion bias are similar across the diversity of prokaryotes, the global mean values of these factors can be used in the model. Under this assumption, the different observed mean genome sizes among prokaryotic groups are due to the differences in the effective population sizes (*N*_*e*_). The model then predicts a global trend curve, which represents the dependency of the mean genome size on the effective population size. More realistically, however, the selection coefficients and the deletion bias values can differ between prokaryotic groups, and the observed genome sizes therefore deviate from the global trend. The challenge is to account for such deviations as fully as possible, without discounting the effect of the universal behavior.

In our previous study [10], the data were fitted to the model in two stages: first, the global parameters were fitted, and at the second stage, some parameters were taken as latent variables and were optimized to maximize the log-likelihood. This methodology is most accurate when deviations from the global trend are small compared to the distribution width. Here, we substantially modify the fitting procedure, to account for the specific factors affecting the genome evolution in different groups of prokaryotes, without obscuring the global trend. The resulting parameters of microbial evolution appear to be more realistic than those obtained with the previous, simplified approach.

## Results

### The workflow and genomic data

**Fig 1:**
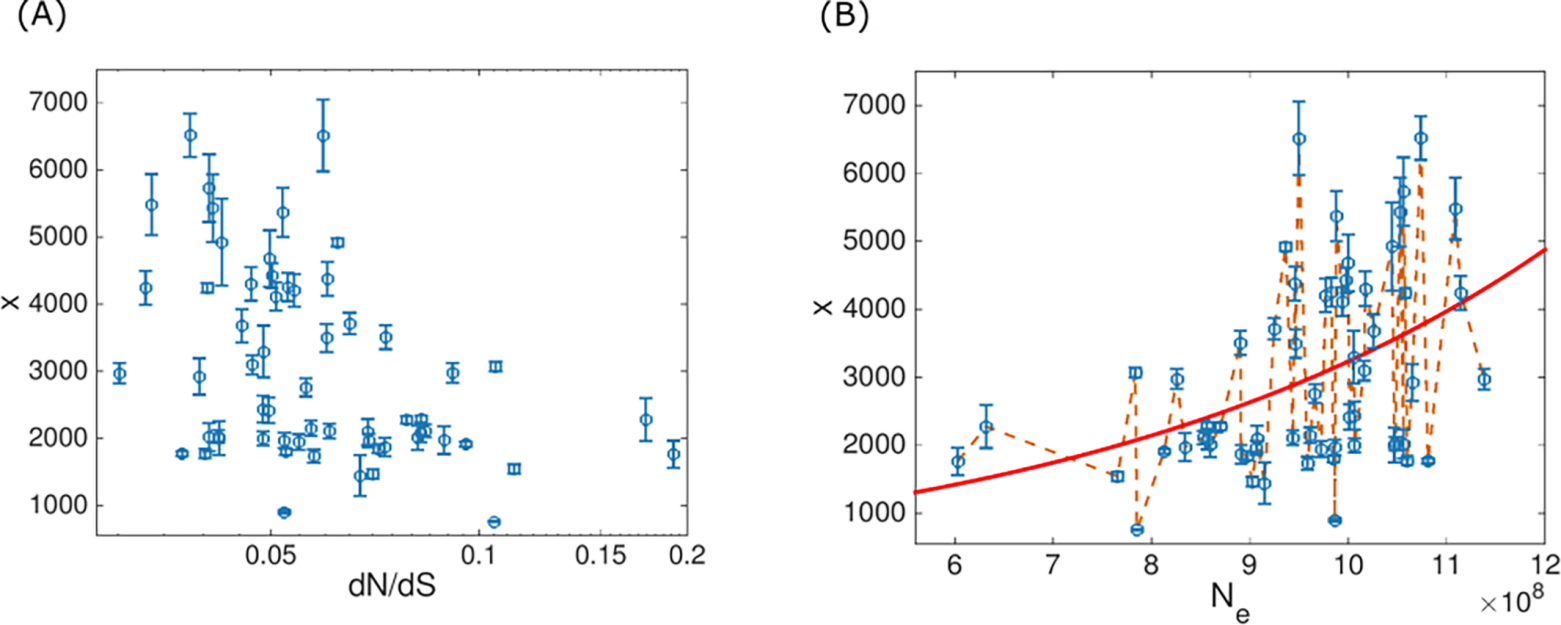
Genome size and selection strength in prokaryotes. (**A**) Mean number of genes *x* is plotted against inferred selection strength d*N*/d*S* where each point represents one prokaryotic cluster (ATGC). Error bars represent genome sizes distributions widths and indicate one standard deviation. (**B**) Mean number of genes is plotted against extracted effective population size *N*_*e*_. A representative global trend curve of mean genome size as predicted by the model (see Eq (7)), where all model parameters are assumed to be global ***θ*** = {*s*,*r*′,*λ*} is indicated by a red line. The approach implemented in the hard fitting methodology, where Eq (7) is used in order to set latent variable value such that model distributions are centered around observed genome sizes, is illustrated in a dashed orange line.

This work extends our previous theoretical analysis of prokaryotic genome evolution and is tightly linked to that study [10]. Accordingly, in what follows, we briefly describe the main result of the previous analysis (Fig 1), including description of the genomic data set, applied methodologies and mathematical modeling framework. The objective is to infer from the data model parameters, which describe the mean deletion bias and selection coefficient that are associated with a single gene gain. Next, we present the general maximum likelihood framework, which is used to optimize model parameters to fit the data. Finally, we develop and apply two fitting methodologies to infer from the data optimal, lineage-specific model.

A data set of 707 bacterial and archaeal genomes clustered in 60 groups of closely related organisms was constructed using the Alignable Tight Genomic Cluster (ATGC) database [18,19]. In the ATGCs, genomes are grouped based on the conservation of orthologous gene sequences and local gene order. In addition to the genome size, which is known for all species in the database, a characteristic value of selection strength was assigned to each cluster (see Fig 1A and Materials and Methods for more details). The effective population size *N*_*e*_ for each cluster was then deduced for each ATGC from the typical associated selection strength (see Fig 1B), using the approach of Kryazhimskiy and Plotkin [20].

### Global model of genome evolution

The mean genome sizes and the d*N*/d*S* values correlate negatively and significantly, with the Spearman’s rank correlation coefficient *ρ* = −0.397 and *p*-value 0.0017, in agreement with the previous observations [8–10](Fig 1A). Effective population sizes are extracted from the d*N*/d*S* values for each ATGC, resulting in the same correlation, but with the opposite sign, between genome size *x* and *N*_*e*_. These correlations indicate that the genome size is determined, to a large extent, by global evolutionary factors that are shared by all prokaryotes. On top of the global factors, there obviously are local influences, such as different lifestyles, environments and availability of genetic material. The goal of the present work is to accurately assess the global factors that govern genome size evolution and are partially masked by local effects, and additionally, to compare the local factors for different groups of bacteria and archaea.

Evolution of prokaryotic genomes can be described within the framework of population genetics by a stochastic process of gene gain and loss events [10]. In brief, a genome is modeled as a collection of *x* genes, where genome size is assumed to evolve through elementary events of acquisition or deletion of one gene at a time. These acquisition or deletion events affect the fitness of the organism, which is assumed to be a function of genome size *x* only. Acquisition and deletion events occur with rates *α* and *β*, respectively. Genes are assumed to be acquired from an infinite gene pool. Gene gains and losses are either fixed or eliminated stochastically, with a fixation probability *F*. In the weak mutation limit, the fixation probability can be expressed as [21]

$$F(s)=\frac{s}{1-e^{-N_{e∙}s}}$$ [1]

where *N*_*e*_ is the effective population size and *s* is the selection coefficient associated with acquisition of a single gene. That is, assuming that the reproduction rate for genome of size *x* is 1, the reproduction rate for a genome of size *x* + 1 is 1 + *s*. To obtain the selection coefficient associated with deletion of a gene, the event of gene deletion is considered: the reproduction rate for genome size *x* + 1 is set as 1, and the reproduction rate for genome size *x* can be therefore approximated by 1 − s, so that

$$s_{\mathrm{deletion}=-}s_{\mathrm{acquisition}}$$ [2]

It should be emphasized that the relation in Eq (2) stems from the single assumption, i.e. that the fitness landscape is a function of genome size only. The gain rate, *P*_+_, is given by the multiplication of the acquisition rate *α*, and the fixation probability of a gene acquisition event. In general, both the acquisition rate and the selection coefficient associated with the acquisition of a gene depend on the genome size:

$$P_{+}(x)=\alpha (x)∙F(s(x))$$ [3]

Using the relation *s*_deletion_ = −*s*_acquisition_ derived above, we get a similar expression for the loss rate, denoted by *P*_

$$P_{-}(x)=\beta (x)∙F(-s(x))$$ [4]

Genome size dynamics is then a chain of stochastic gain and loss events, and can be described by the equation

$$\dot{x}=P_{+}(x)-P_{-}(x)$$ [5]

If for a some value of *x*, denoted *x*_0_, gain and loss rates are equal, i.e. the evolving genome fluctuates stochastically around this value (under a condition discussed below, see Eq (9) below), the dynamics of Eq (5) implies a steady state distribution *f*(*x*) of the genomes sizes. This distribution has an extremum at *x*_0_, and is given by (see Materials and Methods for derivation)

$$f(x)\propto {\left[P_{+}(x)+P_{-}(x)\right]}^{-1}{∙e}^{2\int^{x}\frac{P_{+}(u)-P_{-}(u)}{P_{+}(u)+P_{-}(u)}du}$$ [6]

**Fig 2:**
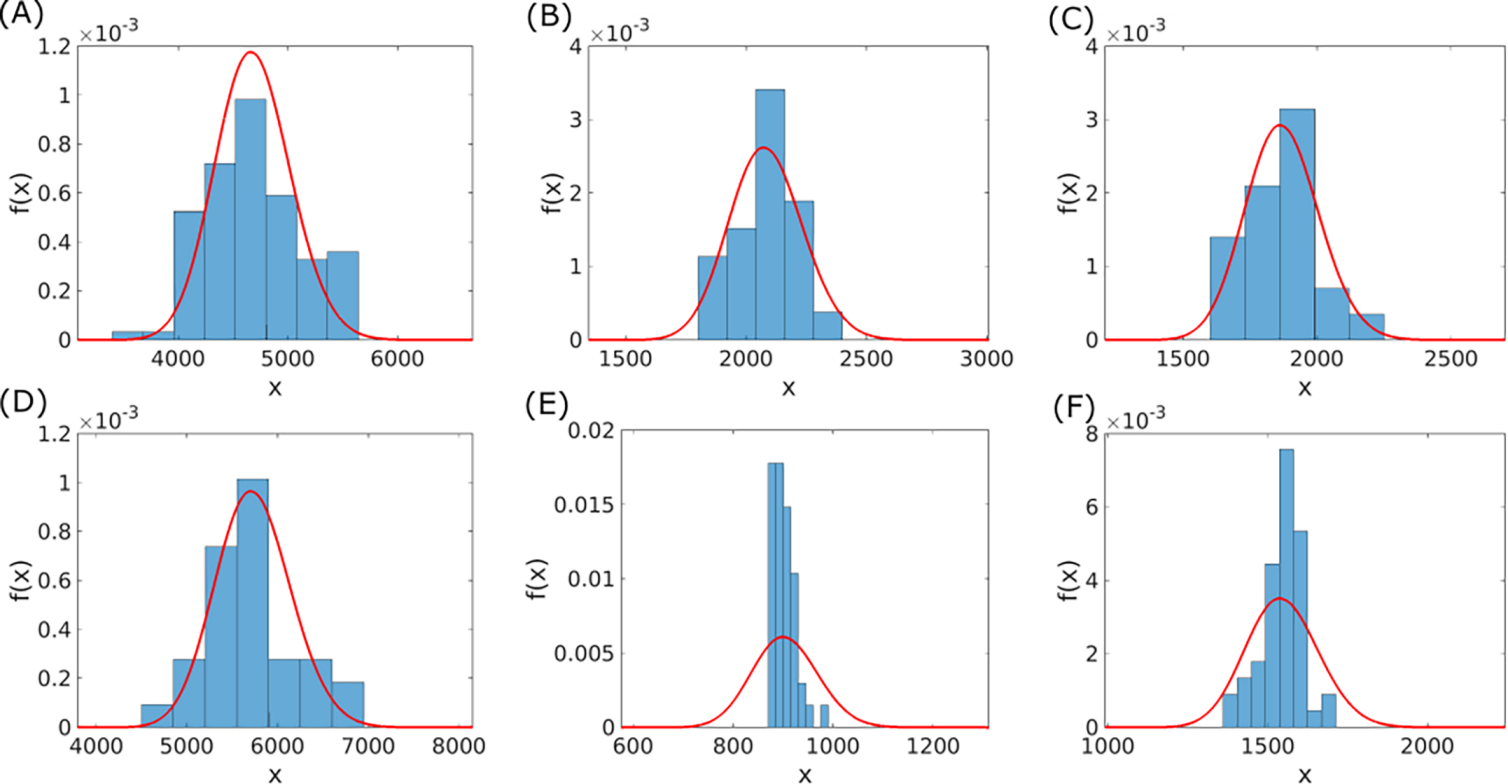
Comparison of the observed and model-generated genome size distributions for 6 ATGCs that consist of at least 20 species. Empirical genome sizes are indicated by bars and model distributions by red solid lines. For model distributions Eq (6) was used, together with the deletion bias of Eq (16). Model parameters were optimized using the mixture model method, with the linear coefficient *a* of the acquisition rate (see Eq (14)) as latent variable. Optimized parameters are listed in Table 2 and in S2 Table. The ATGCs are as follows (the numbers of genomes for each ATGC are indicated in parentheses): (A) ATGC0001 (109), (B) ATGC0003 (22), (C) ATGC0004 (22), (D) ATGC0014 (31). (E) ATGC0021 (45) and (F) ATGC0050 (51).

If the distribution is symmetric, *x*_0_ is the mean genome size, and given that *f*(*x*) is only slightly skewed with relevant model parameters (see Fig 2), *x*_0_ is taken as an approximation for the mean genome size. With respect to the model parameters, *x*_0_ satisfies the relation

$${r(x_{0})=e}^{N_{e}∙s(x_{0})}$$ [7]

where *r*(*x*) is the deletion bias, defined as the ratio of the deletion and acquisition rates:

r(x)=β(x)/α(x) [8]

The extremum point of *f*(*x*) at *x*_0_ can be either a maximum or a minimum. The case where *f*(*x*) has a minimum at *x*_0_ corresponds to genomes that are either collapsing or growing infinitely, and is biologically irrelevant. The extremum point at *x*_0_ is a maximum when

$$P_{+}^{\prime }(x_{0})<P_{-}^{\prime }(x_{0})$$ [9]

Finally, explicit functional forms for *s*(*x*), *α*(*x*) and *β*(*x*) are assumed in the fitting process. The selection coefficient is taken as constant with respect to genome size

s(x)=const [10]

and two forms of acquisition and deletion rates are considered. The first corresponds to the deletion bias in the form of a power law

$$\alpha (x)=x^{\lambda_{+}}$$ [11]

$$\beta (x)=r^{\prime }x^{\lambda_{-}}$$ [12]

with

$$r(x)=r^{\prime }x^{\lambda }$$ [13]

where *λ* = *λ*_−_ − *λ*_+_; because the distribution given by Eq (6) is not sensitive to *λ*_+_ values, it was set to the value of 10^−3^. In addition, a linear model was considered, where

α(x)=a∙x+b [14]

β(x)=x [15]

and the deletion bias is then given by

$$r(x)=\frac{x}{a∙x+b}$$ [16]

The selection coefficient was taken as constant (independent of genome size) for simplicity. Preliminary calculations with additional linear term in genome size (which in principle can be either negative or positive) gave similar results, both in terms of the log likelihood and fitted parameter values (see S1 Table). Importantly, the sign of the selection coefficient is not assumed *a priori*, but rather, results from optimizing the population model to fit the genomic data. The value of the selection coefficient represents the average selective advantage (for positive *s*) or disadvantage (for negative *s*), which is associated with the acquisition of one gene, when averaging is performed over genes, genomes, environments and time. The deletion bias is modelled by a power law with respect to genome size because it encompasses the two extreme cases of constant or linear dependence, along with all intermediates. For compatibility with birth-death-transfer models, in which linear acquisition and deletion rates are assumed [22], the deletion bias given by Eq (16) was studied as well. In this analysis, there is no assumption that of a deletion bias [*r*(*x*) > 1]. The deletion bias value is an outcome of the fitting of the model to the genomic data. With the formulations given above, the population model for genome size evolution contains one known parameter, *N*_*e*_, and a set of three unknown parameters: either {*s*,*r*′,*λ*} or {*s*,*a*,*b*}, depending to the choice of the model for the acquisition and deletion rates.

### Group-specific factors in prokaryotic genome evolution

**Fig 3:**
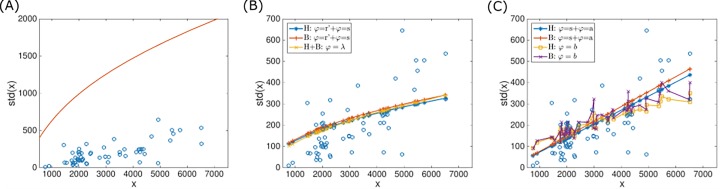
Prokaryotic genome size distribution width plotted vs. genome size. The standard deviation is taken as the proxy for the distribution width. ATGCs are indicated by circles and model fits by lines. (A) Model prediction using the deletion bias of Eq (13) with parameters optimized under the assumption that all three model parameters as universal [10]. (B) Six model fits with the deletion bias of Eq (13) (fitted parameters are given in Table 1). In all fits, one model parameter was set as a latent variable. The model parameter that was set as a latent variable and the methodology used for fitting are indicated in the inset; fits that were visually indistinguishable are represented by the same line. H, hard fitting method; B, mixture model. (C) Same as panel B, for the deletion bias of Eq (16) (fitted parameters are given in Table 2).

The assumption that all model parameters are universal across the diversity of prokaryotes translates into a global trend curve (see Fig 1B) because in this case, groups of prokaryotic species differ from each other only by the typical effective population size. However, when the model parameters are fitted under the assumption that all unknown parameters are universal, the distributions predicted by the model are much wider than the observed distributions of the microbial genome sizes (see Fig 3A) indicating that ATGC-specific factors play a non-negligible role in genome evolution. Deviations from the global trend curve due to local effects can be incorporated into the model by introducing a latent variable ***φ***, i. e. assigning ATGC-specific values to one of the model parameters. The underlying assumption is that the universal dependency of the genome size on the effective population size is captured by the global parameters ***θ***, whereas the deviations from the universal behavior caused by ATGC-specific effects are incorporated in the model through different values of a latent variable ***φ***. Because variation in one parameter of the model can be compensated by variation in a different parameter (e.g. the *s* value can be adjusted to compensate for variation in *r*′ resulting in the same distribution; see S1 Fig), standard methods for latent parameters fitting are not applicable. A proper fitting scheme in this case will not regard the different ATGCs as independent, but rather will allow incorporation of a latent variable without compromising the global trendline across the different ATGCs. The comparison of different ATGCs, with different effective population sizes, is a crucial ingredient in the fitting schemes presented below. Consideration of different ATGCs provides for an additional constraint, thus enabling disentanglement of the different model parameters.

Accordingly, we developed two fitting methodologies: i) an *ad hoc* hard-fitting algorithm, which incorporates into the optimization scheme the resulting global trend curve, and ii) mixture model fitting procedure that assumes a prior distribution for the latent variable. In both methodologies, ATGC-specific fixed ***φ*** values are assigned according to the ***θ*** values. The probability of the observed genome sizes, *P*_*o*_(***X***|***θ***,***φ*,*Z***), is calculated numerically using the steady state genome size distribution *f*(*x*) of Eq (6), as explained below. It is assumed that the genomes in each ATGC are sufficiently diverged, such that enough acquisition and deletion events have occurred to explore the relevant genome size range. Under the assumption of a steady state, the relevant range is spanned by the steady state genome size distribution. The stochastic nature of the dynamics assures that, after sufficiently many time steps, genome size can attain any value that has non-zero probability, regardless of the starting point. A lower bound for the number of acquisition and deletion events can be drawn by counting the number of singleton genes in each genome. We verified that the number of singleton genes is sufficiently large in all genomes to justify this assumption [10].

The distributions produced by the model under optimized parameters are compared to the observed distributions in each ATGC, as shown for 6 ATGCs in Fig 2. It should be noted that the population model accounts for genome size distribution within an individual ATGC, where evolution factors are similar for all genomes, and should not be confused with the overall genome size distribution among prokaryotes [3,23]. In the first step of the optimization, latent variable values are set for each ATGC, such that values are assigned to all three unknown model parameters. The details of this stage are discussed below. For each ATGC, acquisition and deletion rates are then calculated, using either Eqs (11) and (12), or Eqs (14) and (15). Together with the fixation probability, which is given by Eq (1) and calculated using the ***θ*** and ***Z*** values, the acquisition and deletion rates are used to calculate the gain and loss rates of Eqs (3) and (4). The gain and loss rates are then substituted into Eq (6), and the genome size distribution is calculated and normalized numerically. Finally, the probability of the observed genome sizes is given by the product of the distribution values at the observed genome sizes ***X***

$$P_{o}(X|\theta ,\varphi ,Z)=\prod_{i=1}^{60}\prod_{j=1}^{M_{i}}P_{o}(x_{ij}|\theta ,\varphi_{i},Z_{i})$$ [17]

where *x*_*ij*_ is observed genome size for species *j* out of *M*_*i*_ species of ATGC *i*, and *φ*_*i*_ and *Z*_*i*_ are ATGC-specific values of the latent variable and effective population size, respectively. For example, when setting the linear coefficient *a* of the acquisition rate of Eq (14) as the latent variable, we have

θ={s,b} [18]

φ=a [19]

$$Z=N_{e}$$ [20]

For given *s* and *b* values, an ATGC-specific value is assigned for *a*, such that values are assigned to all model parameters and *P*_*o*_(***X***|***θ***,***φ*,*Z***) can be calculated following the steps described above.

In the *ad-hoc* fitting procedure, one model parameter is set as a latent variable, and the two remaining unknown model parameters are considered global and included in ***θ***. Eq (7) is used to adjust the latent variable value according to the ***θ*** values such that the mean genome size in the model is the same as mean genome size in the data (see Fig 1B)

φ=φ(θ,X,Z) [21]

The log-likelihood is then calculated using Eq (32) (see Materials and Methods) with

$$P_{\theta }(X|\theta ,Z)=P_{o}(X|\theta ,\varphi (\theta ,X,Z),Z)$$ [22]

and *P*_*o*_(***X***|***θ*,*φ***(***θ***,***X***,***Z***),***Z***) is calculated using Eq (6) as explained above. However, different values of global parameters ***θ*** can be compensated by the value of the latent variable ***φ*** to yield similar genome size distributions (see S1 Fig). Therefore, an additional constraint is applied to the ***θ*** values in the optimization procedure and combined with the log likelihood l(θ) (see Materials and Methods). The global parameters ***θ*** represent the universal evolutionary factors that entail the observed genome size and effective population size correlation. It is therefore natural to use in the optimization not only the log-likelihood but also the goodness of fit of the global trend curve associated with the ***θ*** values. The global trend is produced using Eq (7) by assuming that all three model parameters are universal; however, under this optimization methodology, ***θ*** is a set of only two global model parameters. The set of global parameters ***θ*** is therefore completed by a single representative value of the latent variable, denoted 〈*φ*〉, to produce the global trend curve. The goodness of fit is then given by the *R*^2^ value for the global trend curve and mean genome sizes of the different ATGCs (see Fig 1B). The *R*^2^ value clearly depends not only on the values of the two universal model parameters ***θ***, but also on the value of 〈*φ*〉. For the optimization of ***θ*** values, the maximum possible *R*^2^ value for the given ***θ*** values is taken.

**Fig 4:**
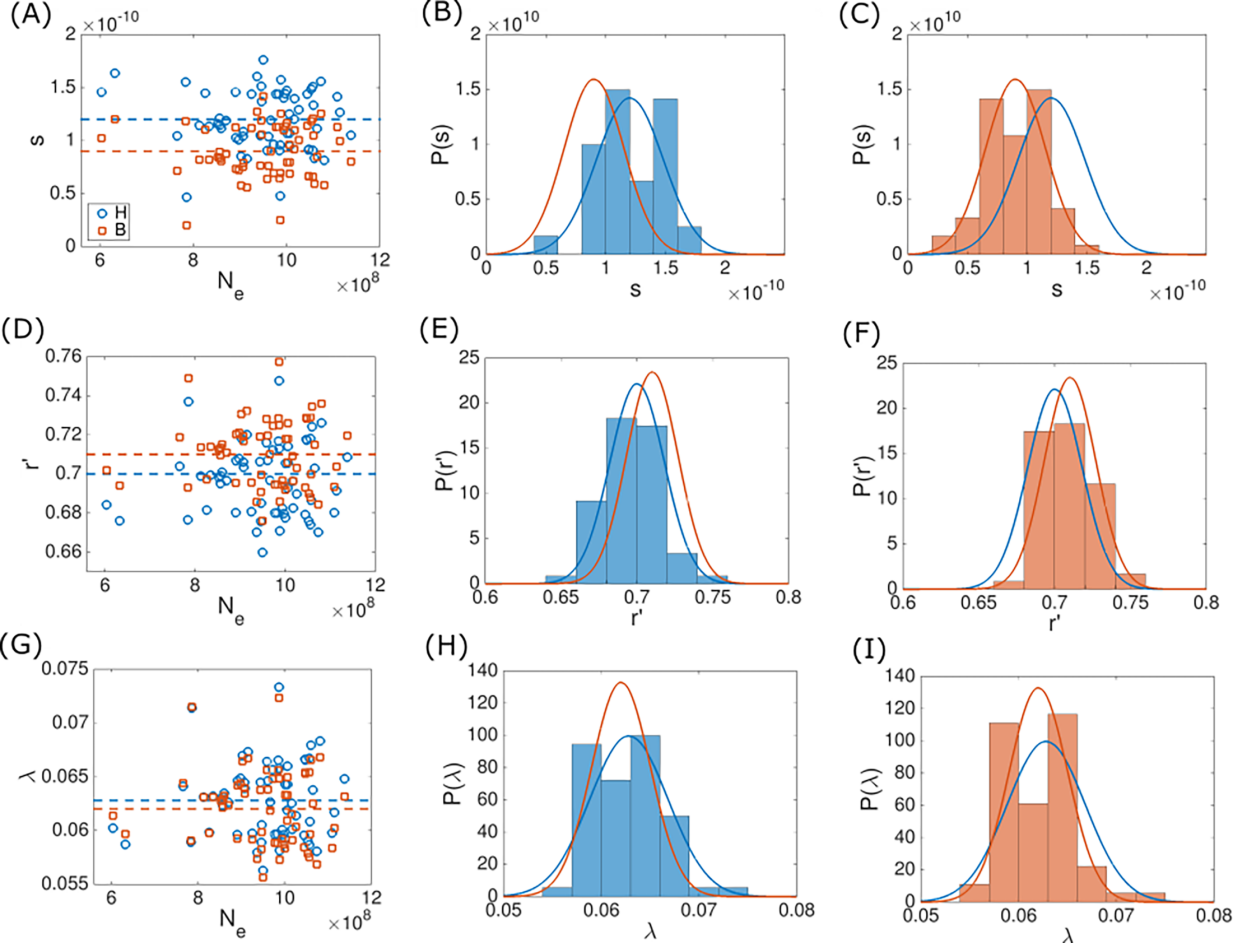
**Fitted latent variable values under the power law deletion bias model (Eq (13)) for *φ* = *s* (A–C), *φ* = *r*′ (D–F) and *φ*** = ***λ* (G–I)**. The fits were obtained using the hard fitting methodology (blue) and the mixture model (orange). Fitted *φ* values for all ATGCs are plotted against the effective population size in the leftmost column. The mean values of the distributions are indicated by dashed lines. The fitted *φ* values histograms are shown together with the latent variable distributions, which are indicated by solid lines. The distribution parameters are given in Table 1. Histograms obtained using the hard fitting methodology are shown in the middle column, and histograms obtained under the mixture model are shown in the rightmost column.

**Fig 5:**
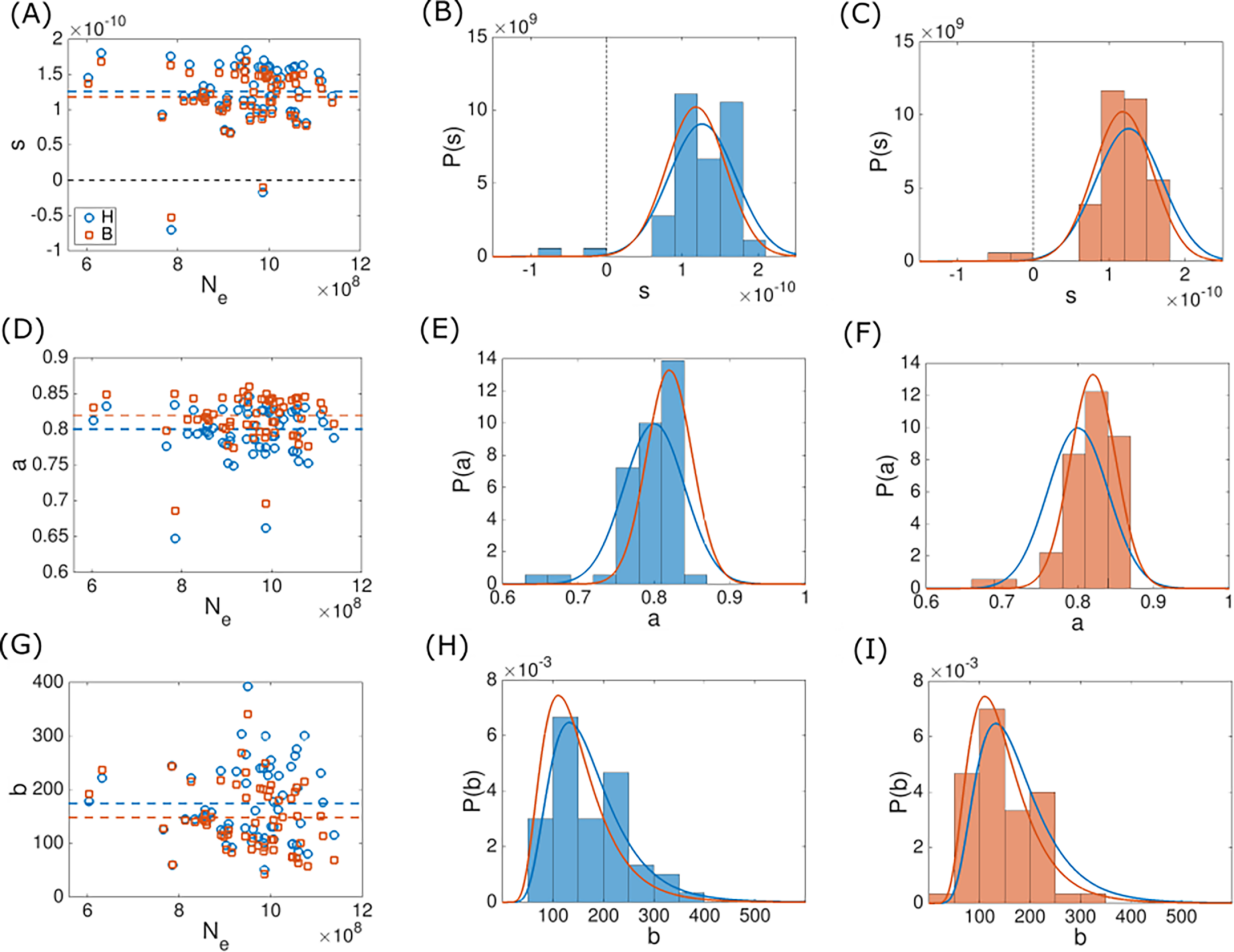
**Fitted latent variable values under the linear deletion bias model (Eq (16)) for *φ* = *s* (A–C), *φ* = *a* (D–F) and *φ*** = ***b* (G–I).** The fits were obtained using the hard fitting methodology (blue) and the mixture model (orange). The fitted *φ* values for all ATGCs are plotted against the effective population size in the leftmost column. Values are indicated by markers and mean values of the distributions are indicated by dashed lines. Fitted *φ* values histograms are shown together with latent variable distributions, which are indicated by solid lines. The parameters of the distributions are given in Table 2. Histograms obtained using the hard fitting methodology are shown in the middle column, and histograms obtained using the mixture model are shown in the rightmost column.

The goodness of fit for the global trend curve is optimized together with the log likelihood, by minimizing a goal function *G*(***θ***):

$$G(\theta )=-l(\theta )/\left|l_{0}\right|-R^{2}(\theta )/R_{0}^{2}$$ [23]

where the log-likelihood and goodness of fit are normalized to give comparable values. Specifically, the values $|l_{0}|=4773$ and $R_{0}^{2}=0.1793$ were used as these are close to the optimal values of log-likelihood and goodness of fit, respectively, for our data set. Fitting was performed for all three assignments of the latent parameter and the two representations of the deletion bias, namely, *φ* = *s*, *φ* = *λ* and *φ* = *r*′ for the deletion bias of Eq (13), and *φ* = *s*, *φ* = *a* and *φ* = *b* for the deletion bias of Eq (16). In all 6 cases, the results were similar, in terms of both the optimized values of the selection coefficient and log-likelihood. The results are summarized in Tables 1 and 2, and the fitted latent variable values are shown in Figs 4 and 5. Notably, there was no significant correlation of the fitted latent variable values and effective population size (Tables 1 and 2), suggesting that the universal correlation between the genome size and the effective population size is not masked by assigning ATGC-specific value to model parameters using this approach. For comparison with the mixture model approach (see below), the optimized latent variable values for all cases but *φ* = *b*, were fitted to a normal distribution. For *φ* = *b*, the fitted values formed a long-tailed distribution (Fig 5) and were accordingly fitted to a log-normal distribution. Fitting was performed by assuming that fitted fixed *φ*_i_ values are samples drawn from a normal distribution with mean *φ*_0_ and standard variation *σ*_*φ*_ (for *φ* = *b*, it was assumed that ln(*φ*) is drawn from a normal distribution)

$$\varphi_{i}∼N(\varphi_{0},\sigma_{\varphi })$$ [24]

where *φ*_0_ and *σ*_*φ*_ were optimized by maximizing

$$l(\varphi_{0},\sigma_{\varphi })=\log[\prod_{i=1}^{60}P(\varphi_{i}|\varphi_{0},\sigma_{\varphi })]$$ [25]

and *P*(*φ*_*i*_|*φ*_0_,*σ*_*φ*_) was calculated using a normal distribution. To assess the fit quality, normality test was performed for (*φ*_*i*_ − *φ*_0_)/*σ*_*φ*_ using the Kolmogorov-Smirnov test against standard normal distribution, with mean 0 and standard deviation 1 (the log of fitted values were tested for normality for *φ* = *b*). For all cases, the null hypothesis that the optimized fixed *φ*_*i*_ values are drawn from a normal distribution could not be rejected. The fitted normal distributions are shown in Figs 4 and 5, and the normal distributions parameters and Kolmogorov-Smirnov test *p* −values are given in Tables 1 and 2.

**Table 1:** Optimal fits for the genome evolution model parameters using the power law model of deletion bias (Eq (13)).

Methodology	*Φ*	*s*	*r*′	*λ*	l(θ)	*R*^2^	KS *p*-value	*φ*_0_	*σ*_*φ*_	*ρ*	*ρ* *p*−value
H	*s*	-	0.693	0.061	−4782	0.179	0.35	1.20 ∙ 10^−10^	2.8 ∙ 10^−11^	−0.06	0.67
B			0.703	0.056	−4975	-	-	9.0 ∙ 10^−11^	2.5 ∙ 10^−11^	0.04	0.78
H	*r*′	1.25 ∙ 10^−10^	-	0.061	−4782	0.179	0.35	0.70	0.018	0.03	0.83
B		1.01 ∙ 10^−10^		0.056	−4975	-	-	0.710	0.017	−0.02	0.87
H	*λ*	1.27 ∙ 10^−10^	0.688	-	−4770	0.179	0.32	0.0628	0.004	0.03	0.80
B		8.7 ∙ 10^−11^	0.666		−4924	-	-	0.062	0.003	−0.1	0.42

**H**, hard fitting methodology; **B,** mixture model fitting.

**Table 2:** Optimal fits for the genome evolution model parameters using the linear model of deletion bias (Eq (16)).

Methodology	*φ*	*s*	*a*	*b*	l(θ)	*R*^2^	KS *p*-value	*φ*_0_	*σ*_*φ*_	*ρ*	*ρ* *p*−value
H	*s*	-	0.810	186	−4700	0.175	0.52	1.26 ∙ 10^−10^	2.8 ∙ 10^−11^	−0.01	0.92
B			0.825	167	−4913	-	-	1.18 ∙ 10^−10^	2.5 ∙ 10^−11^	−0.04	0.79
H	*a*	1.41 ∙ 10^−10^	-	187	−4696	0.175	0.4	0.80	0.04	−0.02	0.88
B		1.28 ∙ 10^−10^		167	−4909	-	-	0.816	0.03	−0.03	0.79
H	*b*	1.30 ∙ 10^−10^	0.824	-	−4759	0.175	0.35	174	77	0.01	0.92
B		1.91 ∙ 10^−10^	0.782		−4944	-	-	148	68	−0.24	0.06

**H**, hard fitting methodology; **B,** mixture model fitting. For the description of the columns, see Table 1.

In the ad-hoc hard fitting method described above, Eq (7) was used to adjust latent variable values such that the model distributions centered around the observed genome sizes. The fitted latent variable values are then scattered around some typical value (Figs 4 and 5). Moreover, fitted values form distributions that are statistically indistinguishable from normal distributions (with the exception of the case *φ* = *b*, which forms a log-normal distribution). It is possible to rely on this observation and implement an alternative optimization methodology, where a prior distribution *P*_*φ*_ is assumed for the latent variable. In the following, normal distributions were assumed as priors, with the exception of a log-normal distribution for the case when *b* is set as the latent variable. Normal (or log-normal) distribution was chosen because the latent variable values fitted using the ad-hoc methodology form a distribution which is indistinguishable from a normal (or log-normal) distribution (see Tables 1 and 2, and Fig 5). As explained below, the mean and variance of the prior distribution are also optimized in the fitting process, and it is only the shape of the prior distribution that is assumed. Accordingly, a specific value *φ*_*i*_ of the latent variable is associated with a probability *P*_*φ*_(*φ*_*i*_|*φ*_0_,*σ*_*φ*_). The probability of the observed genome sizes *x*_*ij*_ for species *j* of ATGC *i* can be then calculated using the Bayes rule, and is given by [24]

$$P(x_{ij}|\theta ,\varphi_{i},Z_{i},\varphi_{0},\sigma_{\varphi })=P_{o}(x_{ij}|\theta ,\varphi_{i},Z_{i})∙P_{\varphi }(\varphi_{i}|\varphi_{0},\sigma_{\varphi }).$$ [26]

The probability of *x*_*ij*_ depends on the prior distribution of *φ*_*i*_ parameters (*φ*_0_ and *σ*_*φ*_) indirectly: *x*_*ij*_ depends directly on *φ*_*i*_, which in turn occurs with the probability *P*_*φ*_ that depends on hyperparameters *φ*_0_ and *σ*_*φ*_. The prior distribution hyperparameters are optimized as well during the fitting process and are therefore included in the set of global parameters ***θ***. The log-likelihood is then given by l(θ,φ)

$$l(\theta ,\varphi )=\log[\prod_{i=1}^{60}P_{\varphi }(\varphi_{i}|\theta )∙\prod_{j=1}^{M_{i}}P_{o}(x_{ij}|\theta ,\varphi_{i},Z_{i},)]$$ [27]

where *x*_*ij*_ is observed genome size for species *j* out of *M*_*i*_ species of ATGC *i*. In more compact way, the equation above can be written as

$$l(\theta ,\varphi )=\log[P_{o}(X|\theta ,\varphi ,Z)∙P_{\varphi }(\varphi |\theta )]$$ [28]

Under this formulation, the maximization of l(θ,φ) is performed in a 64-dimensional parameter space (60 ***φ*** latent variable values, 2 global model parameters ***θ*** and 2 parameters describing the prior distribution *P*_*φ*_ of the latent variable). However, for the optimization of ***θ***, it is possible to sum over all possible values of the latent variable ***φ***, such that *P*_*θ*_(***X***|***θ***,***Z***) of Eq (32) (see Materials and Methods) is given by

$$P_{\theta }(X|\theta ,Z)=\int d\varphi ∙P_{o}(X|\theta ,\varphi ,Z)∙P_{\varphi }(\varphi |\theta )$$ [29]

and the optimization of ***θ*** is performed by maximizing l(θ). To test the validity of the two fitting methodologies presented here, when applied using the population-genetic model of genome evolution, 9 realizations of artificial ATGCs ware generated using the distribution of genome sizes given by the model (Eq (6); see Materials and Methods for details). The realizations were generated using parameter values similar to the fitted parameters obtained using the hard fitting methodology. We then applied both, the mixture model fitting algorithm and the ad-hoc hard fitting methodology to the artificial ATGCs and verified that the optimized parameters values were similar to those of the parameters used for generating the artificial ATGCs. The results for the mixture model are shown in S2 Fig and the hard fitting results in S3 Fig. In all realizations, the *λ* value was inferred to high accuracy. The fitted values of *s* and *r*′ typically have larger errors because variation of *s* can be compensated by the variation of *r*′, and vice versa. Accordingly, the fitted values of *s* and *r*′ follow a line (Panel D in S2 Fig and Panel B in S3 Fig). Notably, the error percentage for *r*′ is modest, and the correct order of magnitude was retrieved for the *s* value, where the overall range of error is similar for both fitting methodologies. For the mixture model, the under-estimation of *λ* is compensated by slightly greater values of *r*′, resulting in a slight offset of the *s* − *r*′ trend curve with respect to the actual values. Accordingly, a slight over-estimation of *λ*, which is observed for the hard fitting optimization, is translated to a slight offset of the *s* − *r*′ trend curve in the opposite direction. Finally, the mixture model was applied to optimize model parameters according to the genomic data, where one genome size model parameter is set as latent variable. Fitted values of global parameters ***θ*** are summarized in Tables 1 and 2, where global parameters now include the parameters of the prior distribution of the latent variable, *φ*_0_ and *σ*_*φ*_. Using these optimized ***θ*** values together with Eq (27) allows fitting the ATGC-specific fixed ***φ*** values (Figs 4 and 5). As with the ad-hoc hard-fitting methodology, there was no significant correlation between fitted ***φ*** values and *N*_*e*_ (see Tables 1 and 2), with the exception of *φ* = *b*, where the Spearman’s correlation coefficient is *ρ* = −0.24 with *p* –value 0.06. Notably, both optimization methodologies gave similar results in terms of the optimized values of ***θ*** and ***φ***, as shown in Tables 1 and 2, and Figs 4 and 5.

In all cases, the genome size distributions produced by the model centered on the observed genome sizes, either by design, as in the hard-fitting algorithm, or as a result of maximizing the log-likelihood, as in the mixture model. However, the observed widths of the genome size distributions are not predicted perfectly by the model, as shown in Fig 3. It is therefore natural to consider the case where more than one model parameter is set as a latent variable. Although generalizing the mixture model to account for more than one latent variable is straightforward, the calculation of the integral of Eq (29) is computationally intensive for more than one latent variable. However, it is possible to explore a setting with more than one latent variable in the mixture model that is expected to produce similar results. As the calculation of the integral in Eq (29) is computationally expensive, the assessment is performed using the expression for l(θ,φ) of Eq (27). Specifically, for deletion bias modelled as in Eq (13), all three genome size model parameters (*s*,*λ* and *r*′) are set as latent variables, and the normal distributions fitted to the latent variables values obtained by applying the hard-fitting methodology are used as priors. Prior distributions are not optimized such that the product term of Eq (27) can be calculated separately for each ATGC, with high efficiency. It is important to note that this is an approximation because the prior distributions that are used here were obtained when optimizing one latent variable at a time. Another possibility is to perform the optimization in the 64 dimensional parameter space of l(θ,φ) in two stages: for the given ***θ*** values, latent variables are fitted for each ATGC separately such that $l(\theta ,\varphi_{i})$ is maximized. This approach was applied for ***φ*** = {*λ*,*r*′}. Both assessments produced results similar to those obtained for one latent variable, so we conclude that, within the current modeling framework, the agreement between the model and the observed genome size distributions cannot be significantly improved further by considering additional latent variables under the mixture model.

**Fig 6:**
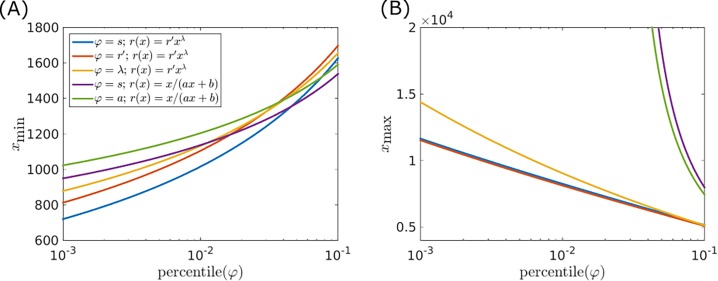
Maximum and minimum equilibrium genome sizes calculated using Eq (7) with parameters fitted under the mixture model. Latent variables and deletion bias models are indicated in the inset. The effective population size was set as *N*_*e*_ = 10^9^. For each fit, the latent variable was taken from the left tail (percentiles 1–10) or the right tail (percentiles 90–99) of the optimized distribution of the latent variable. All estimates for maximum or minimum genome sizes, based on the different choices of the latent variable, are plotted together. As a result the same figure mixes distributions left and right tail for different choices of *φ*. (A) For *φ* = *r*′ and *φ* = *λ* the *x* axis indicates 1 – *P*, where *P* is the percentile. (B) For *φ* = *s* and *φ* = *a* the *x* axis indicates 1 − *P*, where *P* is the percentile.

Finally, the distributions for the latent variable can be used in order to derive estimations for maximum and minimum genome sizes. The optimized ***θ*** values together with *φ* values from the optimized prior distributions tails were substituted into the model approximation for mean genome size of Eq (7). Specifically, *φ* values from percentiles 1 to 10 and 90 to 99 were used, where each of the two ranges corresponds either to the maximum or to the minimum genome size estimates, depending on the choice of the latent variable. For example, when *φ* = *λ* or *φ* = *r*′, the left tail of the distribution (1 to 10 percentile) corresponds to the maximum genome size estimates, whereas for all other choices of *φ*, the left tail corresponds to the minimum genome size estimates. The effective population size was set arbitrarily to *N*_*e*_ = 10^9^. Estimations for *φ* = *s*, *φ* = *λ*, *φ* = *r*′ and *φ* = *a* are shown in Fig 6. For deletion bias modeled by Eq (13), the estimates are roughly consistent with the observed minimum and maximum genome sizes of prokaryotes (excluding the smallest genomes of intracellular parasitic bacteria) [3]. Notably, genome size diverges for the deletion bias of Eq (16) with *φ* = *s* or *φ* = *a* as a latent variable. The deletion bias of Eq (16) results from linear approximations for the acquisition and the deletion rates. Accordingly, gain and loss rates are linear with respect to genome size, where the slope of *P*_+_ is smaller than the slope of *P*_−_, albeit with a non-zero intercept (model parameter *b*). A finite genome size *x*_0_, for which *P*_+_ = *P*_−_ (stationary state), therefore exists, and the condition of Eq (9) is satisfied. In contrast, for $a=e^{-N_{e}s}$, both rates, *P*_+_ and *P*_−_, have the same slope and *P*_+_ > *P*_−_ for all genome sizes, such that the genome size diverges.

Columns: *φ* indicates which model parameter is set as a latent variable; *s*, *r*′ and *λ* indicate global parameters values; l(θ) indicates the log-likelihood of the fit, calculated as detailed in Materials and Method section; *R*^2^ indicates the goodness of fit of the global trend-line and data points as used in the hard fitting methodology (see main text for details); KS *p*-value indicates the *p*-value for rejecting the null hypothesis that the latent variable fitted values distribution is different from a normal distribution, using the KS (Kolmogorov-Smirnov) test; *φ*_0_ is the latent variable normal distribution mean; *σ*_*φ*_ is the latent variable normal distribution standard deviation; *ρ* is the Spearman rank correlation coefficient between fitted latent variable values and *N*_*e*_; *ρ p*−value indicates the significance of the Spearman correlation coefficient *ρ*.

## Discussion

Our previous effort on modeling microbial genome evolution [10] has shown that for all ATGCs, the best fit between the model-generated and observed distributions of genome sizes were obtained with positive *s* values and r>1 (deletion bias). Given that the deletion bias indeed appears to be a universal characteristic of genome evolution [25–27], we have concluded that prokaryotic genomes typically evolve under a selection-mutation balance regime as opposed to a streamlining regime. In biologically oriented terms, these results seem to indicate that, on average, benefits of new genes acquired by microbial genomes outweigh the cost of gene maintenance and expression, conceivably, thanks to the gain of extra metabolic and signaling versatility. However, the actual values of the selection coefficient yielded by the model were extremely low, on the order of 10^−12^, suggesting that the selection affecting an average gene was weak, but also that these values could be under-estimates. The latter possibility was additionally suggested by the observation that, although the model yielded good fits for the means of the genome size distributions, the width of the distributions was significantly over-estimated (Fig 3A). In the previous study [10], we made the strong assumption that the parameters of microbial genome evolution were universal across the entire prokaryotic diversity represented in the ATGCs. The results indicate that the contribution of the universal factors is indeed substantial but fails to account for all or even most of the variation in the genome size distributions indicating that, perhaps not unexpectedly, ATGC-specific factors are important for genome evolution as well. The application of the two methodologies described above significantly improved the agreement between observed and fitted distributions width (Fig 3B and 3C). Notably, all possible combinations of fitting methodologies and latent variables (ad-hoc hard fitting or mixture model combined with either one of model parameters as a latent variable) gave similar results. However, for some ATGCs, the width of the fitted distribution deviates from the observed one (*e*.*g*. ATGC021 and ATGC050 which are shown in Fig 2E and 2F). An over-estimation of genome size distribution width by the model can result from insufficient exploration of genome sizes by genomes in the respective ATGC. Indeed, the currently available genomes represent a sample from the totality of extant genomes, and it seems most likely that, with the growing number of sequenced genomes, the observed genome size distributions become wider.

The two fitting methodologies presented here account for the variability in model parameters across different groups of prokaryotes. However, within each ATGC, a single set of parameters applies to all genes. Actually, however, genes that belong to different functional classes differ in their selective benefit (or cost) [19], acquisition rates [28], and the duration of persistence in the genome [29]. For example, selfish genetic elements are typically associated with proliferation bursts and incur a fitness cost, in contrast to house-keeping genes that are rarely acquired and, being highly beneficial, are even more rarely lost [19][28]. However, under the assumption of a steady state genome size distribution, the differences between the replacement rates of different gene classes are irrelevant for the evolution of the genome size [29]. A single value that represents all genes in the genome can be regarded as an average over all acquisition and deletion events, over time and gene classes.

In the present work, we attempted to take into account the group-specific evolutionary factors by using two independent optimization approaches. Both procedures were used together with two different functional forms of the deletion bias. In all cases, the results were similar, with *s*∼10^−10^, *λ*∼0.06 and *r*′∼0.7 for a power law deletion bias (Table 1), and *s*∼10^−10^, *a*∼0.8 and *b*∼175 for a deletion bias based on linear acquisition and deletion rates (Table 2). It should be stressed that, when optimizing model parameters to fit the data, only partial disentanglement of *s* and *r*′ is achievable, and accordingly, it is the order of magnitude of *s* rather than the actual value which should be taken into account. Introducing latent variables allowed incorporation of ATGC-specific effects into the fitting process. However, variation in one model parameter can be compensated by adjustment of another model parameter, such that all fits are similar in terms of log-likelihood, and thus it is impossible to disambiguate global from local factors affecting the evolution of genome size in terms of model parameters. Nevertheless, the optimized values of the latent variables form relatively narrow distributions around the means (Figs 4 and 5), such that, for the deletion bias of Eq (13), the ratios between standard deviation and mean values are 0.28, 0.06 and 0.03 for *φ* = *s*, *φ* = *λ* and *φ* = *r*′, respectively. For the linear deletion bias given by Eq (16), the ratios between standard deviation and mean values are 0.35, 0.05 and 0.46 for *φ* = *s*, *φ* = *a* and *φ* = *b*, respectively. In both cases, the higher value among those obtained with the hard fitting and the mixture model methodologies is indicated. Thus, the mean values give good estimates for model parameters for all ATGCs. The mean selection coefficient of *s*∼10^−10^ associated with the gain of one gene implies that, on average, acquisition of a gene is beneficial, and that microbial genomes typically evolve under a weak selection regime, with the characteristic selection strength *N*_*e*_ ∙ *s*∼0.1. In highly abundant organisms, transition to a strong selection regime, with *N*_*e*_ ∙ *s* > 1.0, appears possible. It should be noted that *N*_*e*_ ∙ *s* and the deletion bias are invariant to the calibration of *N*_*e*_ that here was based on the assumption of *N*_*e*_ = 10^9^ for ATGC001. These values of *N*_*e*_ ∙ *s* appear to be substantially more realistic than the lower values obtained in our previous study [10], indicating that global and group-specific evolutionary factors synergistically affect microbial genome evolution. This result is consistent with the observed significant, positive correlation between the genome size and selection strength on the protein level and appears intuitive given the diversity of bacterial lifestyles that conceivably drives adaptive gene acquisition. The selective pressure towards larger genomes, manifested in the positive selection coefficients, is balanced by the deletion bias, which is consistently greater than unity. Crucially, this particular form of the mutation-selection balance, whereby the stationary state involves positive selective pressure for gene acquisition being offset by deletion bias, is an outcome of the fitting process and not an assumption of the model (values of *r* for all ATGCs for all fittings are given in S4 and S5 Tables). The opposite situation, whereby selective pressure towards compact genomes is balanced by an insertion bias, is fully compatible with the modelling framework but is inconsistent with the genomic data. Notably, an independent duplication-loss-transfer model of microbial evolution that we have developed recently in order to compare the evolutionary regimes of different classes of genes has yielded closely similar mean values of the selection coefficient [22].

In this work, the deletion bias is considered genome size-dependent and is modelled as a power law or as the ratio of linear approximations for the acquisition and the deletion rates. We found that the best fitted power value is *λ*∼0.06. This value indicates that the genome size dependencies of gene acquisition and deletion rates are generally similar but the deletion rate grows slightly faster with the genome size. This difference, although slight, could put a limit on microbial genome growth. Estimates for minimal and maximal genome sizes were derived using model parameters from the edges of latent variables distributions (percentiles 1% and 99%). The estimations derived using a power law deletion bias were consistent with the observed prokaryotic genome sizes, genome size diverged when considering values from the edges of the distributions together with a linear approximation for the deletion bias. This divergence suggests that the linear approximation for the acquisition and deletion rates holds only locally, and breaks down when a wide range of parameters is considered.

Given the compensation between the *s* and *r’* values, the comparison between the values of these parameters obtained for different ATGCs should be approached with caution. Nevertheless, with this caveat, it is worth noting that the lowest mean values of the selections coefficient were estimated for parasitic bacteria with degraded genomes, such as *Mycoplasma* and *Chlamydia*, whereas the highest values were obtained for complex environmental bacteria with large genomes, such as *Rhizobium* and *Serratia* (S2 and S3 Tables). These differences are compatible with the proposed regime of adaptive evolution of microbial genomes under (generally) weak selection for functional diversification.

## Materials and methods

### Genomic dataset and estimation of selection pressure and effective population size

A dataset of 707 bacterial and archaeal genomes clustered in 60 groups of closely related organisms, referred to as ATGCs, was constructed using the Alignable Tight Genomic Cluster (ATGC) database [18,19]. Genomes are clustered based on the conservation of orthologous gene sequences and local gene order (for a detailed description of clustering criteria see (Kristensen et al., 2017). For simplicity, these individual genomes will be referred to as “species” although many of them represent strains and isolates within the formally described microbial species. For each ATGC, selection strength was inferred on the protein level, by estimating the d*N*/d*S* ratio of 54 core gene families that are common for all or nearly all prokaryotes. Specifically, these alignments of the core proteins constructed using the MUSCLE program [30] were concatenated, converted to the underlying nucleotide sequence alignments, and the d*N*/d*S* ratio was calculated for each species using the PAML software [31]. The characteristic d*N*/d*S* value for each cluster was estimated as the median d*N*/d*S* for all species pairs in the cluster. As shown previously, the median d*N*/d*S* is a stable characteristic of an ATGC that is robust to variations in the set of genome pairs employed for the estimation, and is independent of tree depth within the ATGCs [9]. For each ATGC, the effective population size *N*_*e*_ is deduced from the typical d*N*/d*S* value, using the approach developed by Kryazhimskiy and Plotkin [20] and discussed in detail previously [10]. The effective population size calculation is performed under the following assumptions. Core genes are assumed to evolve under the weak mutation limit regime, where the mutation rate is low such that mutations appear sequentially. In addition, it is assumed that synonymous mutations are strictly neutral, and that the selection coefficient associated with non-synonymous mutations is similar for all core genes in all prokaryotes. It has to be emphasized that the latter assumption is made only for the 54 core gene families that were used for the calculation of the d*N*/d*S* ratio and that are common for nearly all prokaryotes. Finally, the selection coefficient value of non-synonymous mutations is set such that the effective population size for ATGC001, that contains *Escherichia coli* strains, is 10^9^ and the effective population size for all other clusters is calculated accordingly. This arbitrary calibration of *N*_*e*_ will affect the fitted value of *s*, the selection coefficient which is associated with variation in genome size. However, because the population model for genome size evolution depends only on the product *γ* = *N*_*e*_ ∙ *s* (and not on *N*_*e*_ or *s* separately), *γ* is invariant with respect to the calibration of *N*_*e*_ and the deletion bias is invariant as well.

### Derivation of steady state genome size distribution

Following the genome size dynamics of Eq (5), the genome size distribution satisfies the difference equation

$$f(x,t+\Delta t)=f(x,t)(1-P_{+}(x)-P_{-}(x))+f(x-\Delta x,t)P_{+}(x-\Delta x)+f(x+\Delta x,t)P_{-}(x+\Delta x)$$ [30]

Keeping the first two leading terms in a Kramers-Moyal expansion of the master equation above gives the corresponding Fokker-Planck equation [32]

$$\dot{f}\approx -\frac{∆x}{∆t}\partial_{x}[(P_{+}-P_{-})f]+\frac{{\left(\Delta x\right)}^{2}}{\Delta t}\frac{1}{2}\partial_{x}^{2}[(P_{+}+P_{-})f]$$ [31]

The steady state distribution given by Eq (6) is the solution of the second order differential equation which is obtained from Eq (31) for $\dot{f}=0$. Comparison of the analytical steady state distribution of Eq (6) and steady state genome size distributions obtained from simulations of the stochastic dynamics of Eq (5), are shown in S4 Fig.

### Maximum-likelihood framework for model parameters optimization

The objective is to infer the unknown parameters of the genome size model presented below from the genomic dataset. The probability of a set of observations ***X***, namely, observed genome sizes in all species in all ATGCs, is given by a distribution predicted by the genome size population model. The distribution depends on two types of parameters: known parameters ***Z***, and unknown parameters ***θ***. For the genome size population model, the known parameter is the effective population size *N*_*e*_, which is calculated for each ATGC. The unknown parameters are deletion bias (*r)* and selection coefficient (*s*) associated with the gain of a single gene. Simply put, the goal is to optimize ***θ*** by fitting the model distribution to the observed genome sizes in terms of log-likelihood. Optimization is performed by maximizing l(θ) using using Matlab® for simplex multidimensional search in the parameter space where

$$l(\theta )=\log[P_{\theta }(X|\theta ,Z)]$$ [32]

The calculation of *P*_*θ*_(***X***|***θ***,***Z***) from the genome size population model is presented in detail in the Results section.

## Supporting information

S1 Fig Genome size distribution in ATGC001 that contains 109 species, primarily *E*. *coli* strains. The bars show the observed genome sizes histogram. Solid lines show genome size model steady state distribution of Eq (7) with model parameters as indicated in the legend, for the acquisition and the deletion rates of Eqs (11 and 12) (A) and of Eqs (14 and 15) (B).
(PNG)

S2 Fig Artificial ATGCs realizations using the deletion bias of Eq (13). Model parameters optimization was performed using the mixture model methodology. (**A**): Example for one realization of artificial ATGCs. Error bars correspond to one standard deviation. Solid lines indicate the global trend line given by Eq (7), where mean value of latent variable prior distribution is used. Global trend line for actual model parameters used for the realization is indicated by blue line, and the same line with fitted parameters is indicated by a red line. (**B**): Latent variable *r*′ values in the different artificial ATGCs for the same realization that is shown in panel A. Actual values are indicated by blue circles and fitted values are indicated by red x marks. Mean value of the normal prior distribution is indicated by a dashed line. (**C**): Error percentage is shown for fitted ***θ*** values for 9 realizations by box plots. The error is calculated as 100 ∙ (*ξ*_infected_ – *ξ*_actual_)/*ξ*_actual_. (**D**): Scatter plot for fitted *s* and *r*′ values in 9 different realizations. Actual values are indicated by black filled circle.
(PNG)

S3 Fig Artificial ATGCs realizations. The analysis is similar to that in S2 Fig, only. in this case, the hard fitting methodology was used to optimize model parameters. Panels (A) and (B) are the same as panels (C) and (D), respectively, of S2 Fig.
(PNG)

S4 Fig Comparison of analytical genome size distribution with numerical simulations. Genome size evolution was simulated according to the stochastic dynamics of Eq (5) using Gillespie simulation scheme. For each set of parameters histogram of 1000 replicas (blue bars) is shown together with steady state genome size distribution, as calculated using Eq (6) (solid red line). The gain and loss rates of Eqs (11) and (12) were used in the simulations. All simulations started with genome size *x* = 1000 lasted 10^9^ steps, and were performed with *r*′ = 0.7 and *λ*_+_ = 10^−3^. The rest of model parameters that were used are indicated in each panel.
(PNG)

S1 Table Optimal fits for the genome evolution model parameters using the power law model of deletion bias (Eq. (13)) together with a linear selection coefficient *s*(*x*) = *s*_1_ + *s*_2_ ∙ *x*. **H** hard fitting methodology; **B,** hierarchical Bayesian model fitting.
(DOCX)

S2 Table Optimal fits for the genome evolution model latent variables using the power law model of deletion bias (Eq (13)). (CSV)

S3 Table Optimal fits for the genome evolution model latent variables using the linear model of deletion bias (Eq (16)). (CSV)

S4 Table Deletion bias values for all ATGCs for the power law deletion bias (Eq (13)). The deletion bias is calculated using the mean genome size for each ATGC. Column headers indicate the latent variable (*s*, *r*′ or *λ*) and the fitting scheme used–H for the hard fitting methodology and B for the mixture model.
(CSV)

S5 Table Deletion bias values for all ATGCs for the linear deletion bias (Eq (16)). The deletion bias is calculated using the mean genome size for each ATGC. Column headers indicate the latent variable (*s*, *a* or *b*) and the fitting scheme used–H for the hard fitting methodology and B for the mixture model.
(CSV)
