# Supplementary figures and images for: Estimation of universal and taxon-specific parameters of prokaryotic genome evolution
Source: PLoS One. 2018 Apr 13;13(4):e0195571. doi: 10.1371/journal.pone.0195571 (PMC5898727; doi:10.1371/journal.pone.0195571)

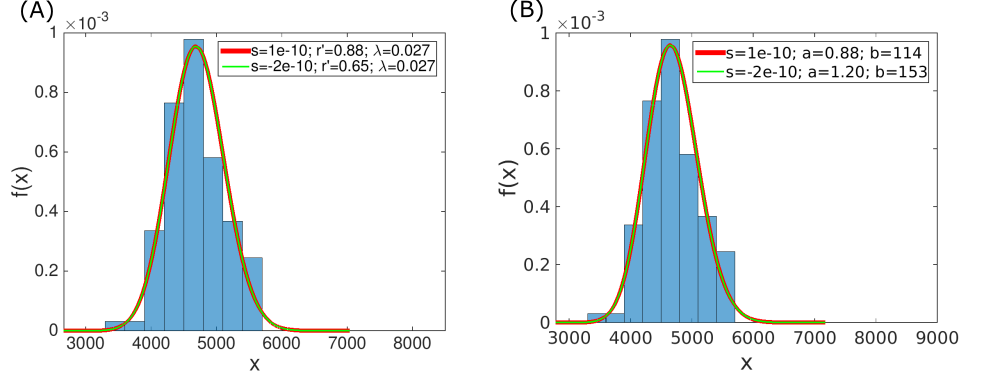

Supplement: S1 Fig — The bars show the observed genome sizes histogram. Solid lines show genome size model steady state distribution of Eq (7) with model parameters as indicated in the legend, for the acquisition and the deletion rates of Eqs (11 and 12) (A) and of Eqs (14 and 15) (B). (PNG) [file pone.0195571.s001.png]

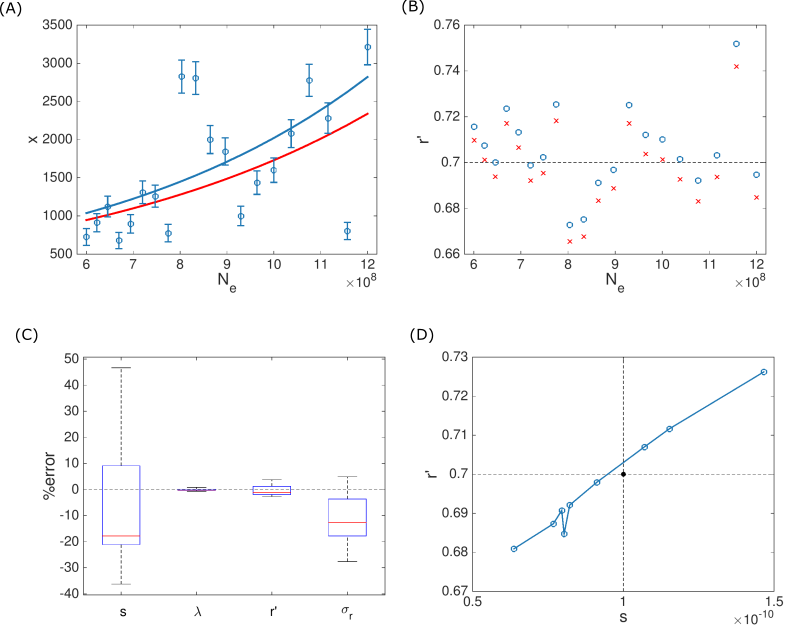

Supplement: S2 Fig — Model parameters optimization was performed using the mixture model methodology. (A): Example for one realization of artificial ATGCs. Error bars correspond to one standard deviation. Solid lines indicate the global trend line given by Eq (7), where mean value of latent variable prior distribution is used. Global trend line for actual model parameters used for the realization is indicated by blue line, and the same line with fitted parameters is indicated by a red line. (B): Latent variable r′ values in the different artificial ATGCs for the same realization that is shown in panel A. Actual values are indicated by blue circles and fitted values are indicated by red x marks. Mean value of the normal prior distribution is indicated by a dashed line. (C): Error percentage is shown for fitted θ values for 9 realizations by box plots. The error is calculated as 100 ∙ (ξinfected – ξactual)/ξactual. (D): Scatter plot for fitted s and r′ values in 9 different realizations. Actual values are indicated by black filled circle. (PNG) [file pone.0195571.s002.png]

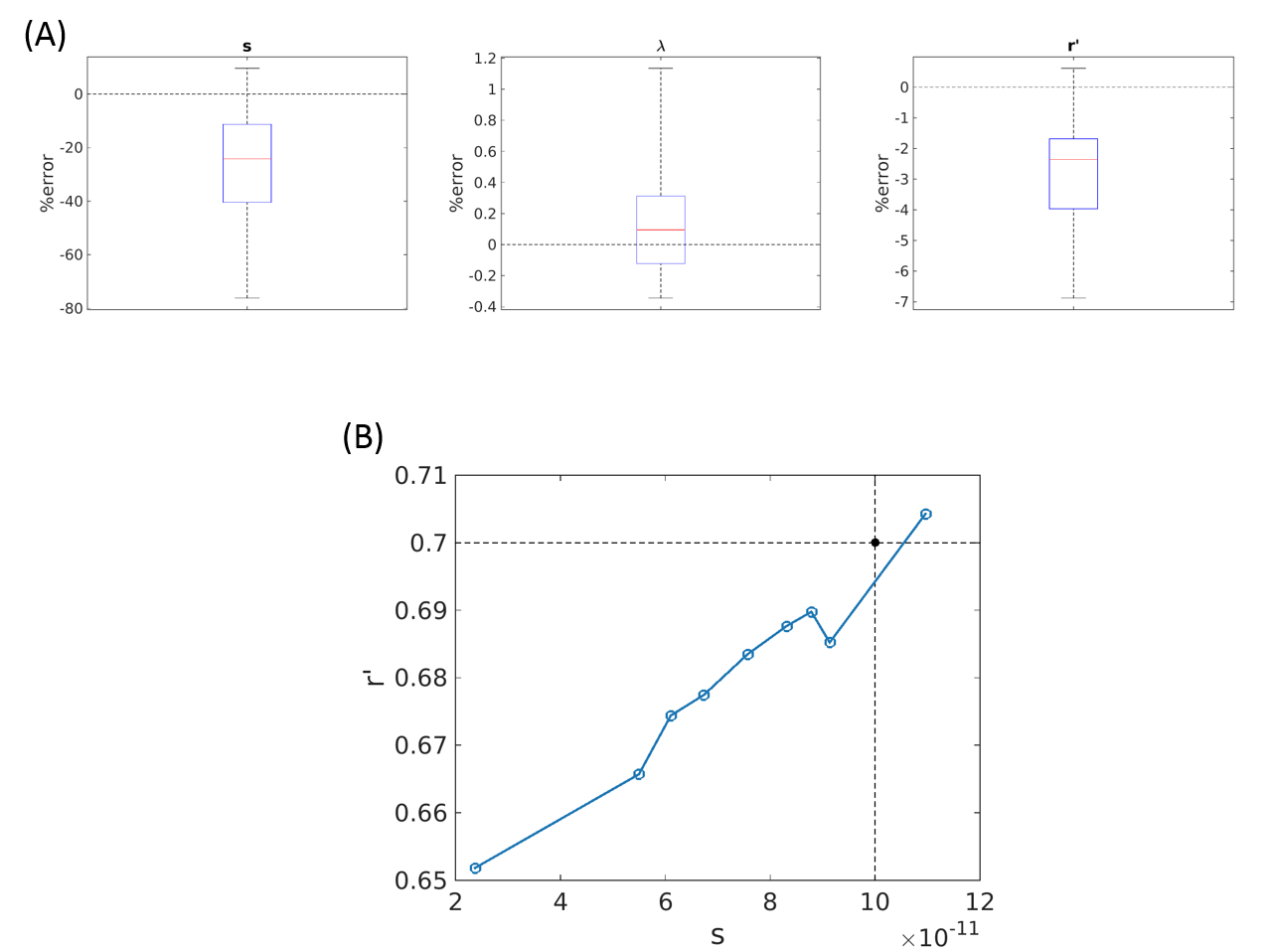

Supplement: S3 Fig — The analysis is similar to that in S2 Fig, only. in this case, the hard fitting methodology was used to optimize model parameters. Panels (A) and (B) are the same as panels (C) and (D), respectively, of S2 Fig. (PNG) [file pone.0195571.s003.png]

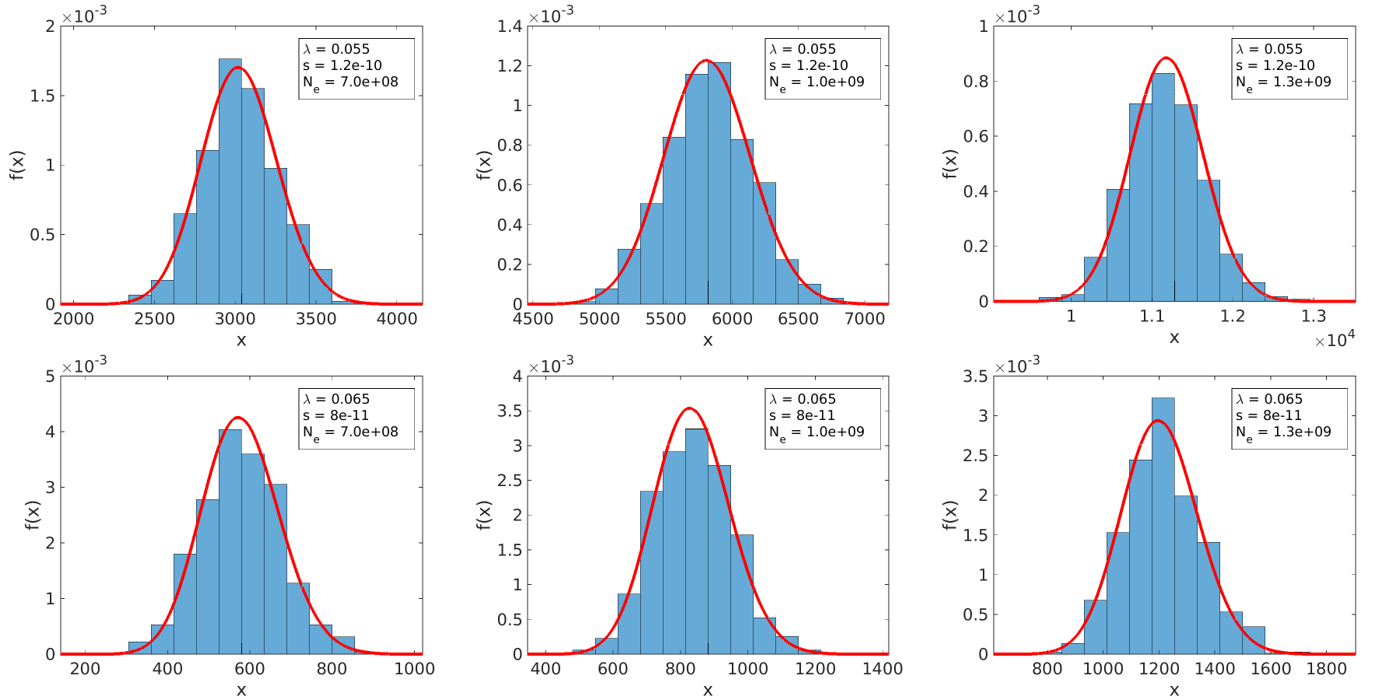

Supplement: S4 Fig — Genome size evolution was simulated according to the stochastic dynamics of Eq (5) using Gillespie simulation scheme. For each set of parameters histogram of 1000 replicas (blue bars) is shown together with steady state genome size distribution, as calculated using Eq (6) (solid red line). The gain and loss rates of Eqs (11) and (12) were used in the simulations. All simulations started with genome size x = 1000 lasted 109 steps, and were performed with r′ = 0.7 and λ+ = 10−3. The rest of model parameters that were used are indicated in each panel. (PNG) [file pone.0195571.s004.png]
