# Supplementary material for: Estimation of universal and taxon-specific parameters of prokaryotic genome evolution
Source: PLoS One. 2018 Apr 13;13(4):e0195571. doi: 10.1371/journal.pone.0195571 (PMC5898727; doi:10.1371/journal.pone.0195571)
Supplement: S1 Table — H hard fitting methodology; B, hierarchical Bayesian model fitting. (DOCX) [file pone.0195571.s005.docx]

| Methodology | $\varphi$ | $s_{1}$ | $s_{2}$ | $\lambda$ | $\mathcal{l}\left( \boldsymbol{\theta} \right)$ | $R^{2}$ | KS $p$-value | $\varphi_{0}$ | $\sigma_{\varphi}$ | $\rho$ | $\rho$  $p-$value |
| --- | --- | --- | --- | --- | --- | --- | --- | --- | --- | --- | --- |
| H | $r^{'}$ | ${1.24\cdot10}^{-10}$ | ${1.27\cdot10}^{-15}$ | $0.066$ | $-4774$ | $0.179$ | $0.34$ | $0.67$ | $0.018$ | $0.03$ | $0.81$ |
| B |  | ${1.28\cdot10}^{-10}$ | ${7.56\cdot10}^{-15}$ | $0.086$ | $-4943$ | - | - | $0.58$ | $0.014$ | $0.11$ | $0.39$ |
